# Sarcopenia in end-stage lung disease patients: a systematic review and meta-analysis

**DOI:** 10.3389/fmed.2025.1640027

**Published:** 2025-10-03

**Authors:** Li Sheng, Peipei Gu, Lingyun Cai, Yan Zhu, Meijun Dong, Fei Zeng

**Affiliations:** Department of Nursing, The Second Affiliated Hospital of Zhejiang University School of Medicine, Zhejiang, Hangzhou, China

**Keywords:** sarcopenia, end-stage lung disease, prevalence, systematic review, meta-analysis

## Abstract

Sarcopenia is usually an age-related disease in the elderly, which also affects patients with end-stage lung disease (ELD), such as chronic obstructive pulmonary disease, interstitial lung disease, and pulmonary hypertension, significantly affects disease progression and clinical outcomes. Currently, there are various methods for quantifying sarcopenia through muscle mass assessment. This study aimed to evaluate the prevalence of sarcopenia in patients with end-stage lung diseases using meta-analysis and to explore its influencing factors and clinical implications. A systematic search was conducted in English databases, including PubMed, Web of Science (WOS), Embase, Cochrane Library, CINAHL, and Scopus. Two researchers independently assessed study quality using the JBI Critical Appraisal Checklist for Analytical Cross Sectional Studies. Data analysis was performed using Stata 18.0 and R 4.4.3 software. The pooled prevalence of sarcopenia in ELD patients was calculated using a random-effects model, with subgroup analyses to identify sources of heterogeneity. Twenty-four cross-sectional studies involving 6,800 patients were included. The pooled prevalence of sarcopenia in ELD patients was 31.6% (95% CI: 26.5–36.8%). Subgroup analyses revealed higher sarcopenia prevalence in patients aged ≥70 years (vs. <70 years), males (vs. Females), those diagnosed using AWGS2019 criteria (vs.other criteria), dual-energy X-ray absorptiometry (DXA; vs. bioelectrical impedance analysis [BIA]), Asian populations (vs. European/North American populations), and COPD patients (vs. IPF/ILD patients). Sarcopenia is prevalent among patients with ELD, with its prevalence varying across different populations due to multiple influencing factors, resulting in distinct clinical outcomes. It is recommended to implement targeted intervention strategies based on these factors to reduce the incidence of sarcopenia and enhance the quality of life.

**Systematic review registration:**

Identifier, CRD420251028682 (PROSPERO).

## Highlights

- Demonstrated a pooled prevalence for sarcopenia in end-stage lung disease patients
- The reasons for the high prevalence of sarcopenia were explained.
- The influencing factors and clinical outcomes associated with sarcopenia in end-stage lung diseases were explored.
- Sarcopenia prevalence varies across populations, necessitating targeted interventions to improve quality of life.

## 1 Introduction

End-stage lung disease (ELD) is a clinical syndrome characterized by progressive, irreversible impairment of pulmonary function and systemic multiorgan pathophysiological disturbances (1, 2). It encompasses life-threatening respiratory conditions such as chronic obstructive pulmonary disease (COPD), pulmonary interstitial fibrosis, and pulmonary arterial hypertension (3). Notably, disease progression is accompanied by complex interacting factors, including chronic hypoxemia, systemic inflammatory cascades, disruption of metabolic homeostasis, and long-term pharmacotherapy (e.g., glucocorticoid use) (4–6), which synergistically drive the progressive depletion of skeletal muscle mass and function—a condition termed secondary sarcopenia. This complication has emerged as a critical, independent risk factor influencing clinical outcomes in ELD patients, profoundly impacting prognosis and therapeutic decision-making (7, 8).

Epidemiological investigations have revealed significant heterogeneity in the prevalence of sarcopenia among patients with ELD, ranging from 7.9 to 89% (8, 9), which is substantially higher than in age-matched healthy populations (7). Sarcopenia exacerbates the progression of primary diseases through multiple pathological mechanisms: it not only directly impairs respiratory muscle efficiency, leading to deterioration in ventilatory function (10), but also induces peripheral muscle atrophy, resulting in a precipitous decline in exercise capacity (11), while significantly increasing the risk of fall-related fractures (12). Clinical cohort studies confirm that ELD patients with comorbid sarcopenia exhibit an elevated risk of hospitalization due to acute exacerbations (13), a prolonged duration of mechanical ventilation dependence (14), and a markedly elevated all-cause mortality rate (15, 16). Given its profound impact on patients’ quality of life and prognosis, early identification and comprehensive intervention for sarcopenia have become critical priorities in multidisciplinary management frameworks for individuals with ELD, underscoring its significant clinical relevance in this field (7, 8, 10).

The diagnostic framework for sarcopenia lacks global consensus, with current diagnostic parameters primarily encompassing skeletal muscle mass quantification (assessed via dual-energy X-ray absorptiometry[DXA] or bioelectrical impedance analysis [BIA]) (17, 18), grip strength measurement, and physical performance evaluation (e.g., gait speed) (17). Recent observational studies have yielded extensive and inconsistent findings (19–21). Notably, the prevalence of sarcopenia appears to be higher in Asian populations compared to those in other regions (22). However, patients with ELD exhibit unique characteristics due to respiratory limitations, frequent acute exacerbations, and complex comorbidities. They may present with distinct phenotypic patterns (e.g., predominant proximal muscle involvement) and pathological mechanisms (e.g., systemic inflammation and hypoxia) that differ from those of other chronic disease cohorts (4, 5, 23). The heterogeneity of ELD etiology, the complexity of disease progression, and the diversity in sarcopenia diagnostic criteria contribute to significant variability in research outcomes. This, in turn, results in fragmented clinical protocols for sarcopenia identification, assessment, and management. Despite the growing body of research on sarcopenia in COPD, idiopathic pulmonary fibrosis, and pulmonary hypertension, critical clinical questions remain unanswered. This knowledge gap severely hampers the development of evidence-based practice guidelines, highlighting the urgent need to establish a multidimensional ELD-specific sarcopenia assessment framework.

Thus, this study utilizes systematic review and meta-analysis methodologies to comprehensively assess the prevalence of sarcopenia among patients with ELD and to ascertain its variability within distinct ELD subgroups. Through the synthesis of existing evidence, our goal is to establish an evidence-based framework for sarcopenia management in ELD, offering clinicians actionable insights to enhance therapeutic strategies.

## 2 Methods

### 2.1 Study registration

The protocol for this systematic review has been registered with the International Prospective Register of Systematic Reviews (PROSPERO; Registration ID: CRD420251028682).

### 2.2 Data source and search strategy

The study was conducted by two graduate students (LS and MJD) who had received evidence-based practice training. They independently performed systematic searches through English-language databases.

The searches covered PubMed, Web of Science (WOS), Embase, Cochrane Library, CINAHL, and Scopus, with the time range spanning from the inception of each database to March 2025. Database-specific search strategies were collaboratively developed and pre-tested for optimization by the research team in conjunction with experienced librarians. The core search terms included Medical Subject Headings (MeSH) and keywords such as: “COPD (chronic obstructive pulmonary disease OR chronic obstructive lung disease OR COAD),” “ILD/IPF (Idiopathic Pulmonary Fibrosis, interstitial lung disease, idiopathic lung disease),” “CF (cystic fibrosis, fibrosis cystic, mucoviscidosis, pulmonary cystic fibrosis, cystic fibrosis pulmonary),” and “PAH (Hypertension, Pulmonary, Pulmonary Hypertension, lung arterial hypertension),” as well as terms related to sarcopenia: “sarcopenias” OR “sarcopenic” OR “muscle loss” OR “muscle mass” OR “muscle waste” OR “muscle wasting” OR “skeletal muscle reduction” OR “muscle weakness” OR “muscular atrophy.” The complete search strategies are detailed in the Supplementary materials.

In addition, manual screening of the reference lists of all retrieved relevant systematic reviews was conducted to identify additional studies for inclusion. The search was restricted to include only peer-reviewed literature written in English (due to the unavailability of translation support). All search results were consolidated and deduplicated using EndNote reference management software (Thomson Reuters, New York, United States), followed by subsequent processing.

### 2.3 Selection criteria

Inclusion Criteria: ① The study population comprises patients with end-stage lung disease aged ≥18 years; ② The study type is a cross-sectional study; ③ The language of the publication is English; ④ The observed indicators include the incidence of sarcopenia in end-stage lung disease, among others; ⑤ When the research content of a journal article overlaps with that of a dissertation, the journal article is adopted. To ensure the methodological rigor of the included literature, gray literature was not included in this study. Exclusion Criteria: ① The study population consists of individuals aged <18 years; ② The incidence of sarcopenia is not mentioned; ③ The study is an intervention study or a review article; ④ The full text of the study is unavailable, or the study is a duplicate publication.

### 2.4 Quality assessment

The quality assessment of the literature was conducted by two reviewers (LS, Peipei Gu) using the JBI Critical Appraisal Checklist for Analytical Cross-Sectional Studies, a tool specifically designed for evaluating the quality of cross-sectional studies. In cases where there were doubts regarding the quality, a third reviewer was consulted for a reassessment. The JBI quality assessment criteria for cross-sectional studies consist of 8 items, with each item being evaluated as “Yes,” “No,” “Unclear,” or “Not applicable” (24, 25). ① Were the criteria for inclusion in the sample clearly defined? ② Were the study subjects and the setting described in detail? ③ Was the exposure measured in a valid and reliable way? ④ Were objective, standard criteria used for measurement of the condition? ⑤ Were confounding factors identified? ⑥ Were strategies to deal with confounding factors stated? ⑦ Were the outcomes measured in a valid and reliable way? ⑧ Was appropriate statistical analysis used? Supplementary Table 1 quality assessment of the included studies.

### 2.5 Statistical analysis

This study utilized Stata 18 and R 4.4.3 software for statistical analysis to ascertain the prevalence of sarcopenia among ELD patients. Heterogeneity across studies was assessed using the I^2^ statistic, which quantifies the proportion of between-study variance in effect sizes (26). When *p* ≥ 0.1 and I^2^ ≤ 50%, indicating negligible heterogeneity, a fixed-effects model was applied; conversely, when *p* < 0.1 and I^2^ > 50%, suggesting significant heterogeneity (27), a random-effects model was utilized for meta-analysis. Subgroup analyses were conducted based on age, gender, diagnosis, geographic region, and disease type. To evaluate the robustness and reliability of the meta-analysis results, sensitivity analysis was performed by sequentially excluding individual studies to examine their impact on overall heterogeneity. Since single-group rate meta-analysis provides descriptive outcomes rather than comparative difference testing, traditional “positive result” determination or statistical significance thresholds (and associated publication bias assessment) are not applicable (28).

## 3 Results

### 3.1 Study selection process

**Figure 1:**
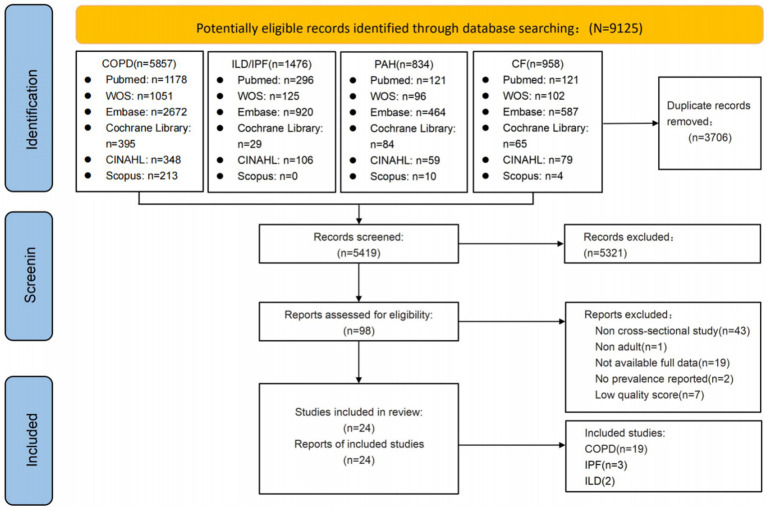
Flowchart of the literature search and selection.

The systematic search across the aforementioned databases initially identified 9,125 relevant articles. After removing duplicates using EndNote 20 software, 3,706 articles remained. Following title and abstract screening, 98 articles were selected for full-text review. After excluding studies with non-conforming study populations, irrelevant topics, and low-quality research, 24 articles were ultimately included in the final selection. Quality assessment demonstrated that all 24 articles were of medium to high quality and were therefore incorporated into the analysis. Given that IPF has distinct pathophysiology, clinical presentation, prognostic implications, and treatment regimen (1), it is routinely classified and studied as a separate entity despite being technically a form of ILD. See Figure 1 and Supplementary Table 1.

### 3.2 Descriptions of the included studies

Among the 24 included studies, 19 studies primarily centered on COPD (10, 22, 25, 29–44), 3 studies focused on IPF (45–47), and 2 studies on ILD (48, 49). Geographically, 17 studies were carried out in Asia (22, 25, 29–31, 34, 35, 37–39, 42, 43, 45–49), 6 studies in Europe (10, 32, 33, 36, 41, 44), and only 1 study in North America (40). Five studies each utilized the EWGSOP2010 (10, 25, 29, 33, 36), EWGSOP2 (35, 40, 41, 43, 44), and AWGS2019 (34, 42, 45, 46, 48) diagnostic criteria, while 3 studies employed the AWGS2014 criteria (22, 37, 39). The diagnostic criteria were not explicitly stated in the remaining studies. In terms of assessment methods, 13 studies used BIA (10, 25, 29, 33, 34, 42–49), and 11 studies used DXA (22, 30–32, 35–41). A summary of the quality scores is provided in Supplementary Table 1, and detailed characteristics of the selected studies are presented in Table 1.

**Table 1:** Characteristics of included studies.

First author	Year	Country	Disease type	Sample size	Age (mean ± SD)	Male, n (%)	Consensus group	Prevalence	Components of sarcopenia					
									Lean muscle mass		Muscle strength		Physical performance	
									Method	Cut-off (kg/m^2^)	Method	Cut-off (kg)	Method	Cut-off (m·s^−1^)/m
Jamie R Chua (25)	2019	Philippines	COPD	41	69.2 ± 6.8	37 (90.2)	EWGSOP2010	46.3%	BIA	M: <8.87F: <6.42	HGS	M: <24.54F: <16.10	6MWD	-
Min Kwang Byun (29)	2017	South Korea	COPD	80	68.4 ± 8.9	67 (83.8)	EWGSOP2010	25%	BIA	M:<8.87F:<6.42	HGS	M:≤30F:≤20	6MWD	-
Jamie R Chua (30)	2020	Philippines	COPD	41	69.2 ± 6.8	-	-	46.3%	DXA	M:<12.50F:<8.33	HGS	M: <24.54F: <16.10	6MWD	-
Jae Ho Chung (31)	2015	South Korea	COPD	1,039	Man: 64.5 ± 9.4 Woman: 64.5 ± 10.2	-	-	27.2%	DXA	M:<6.95F:<4.94	-	-	-	-
Tatiana Munhoz (32)	2015	Basil	COPD	91	67.4 ± 8.7	-	-	39.6%	DXA	M:<7.26F:<5.45	-	-	6MWD	
Francesca de Blasio (33)	2018	Italy	COPD	263	68.0 ± 9.0	-	EWGSOP2010	24.0%	BIA	M:≤8.50F:≤5.75	HGS	M:<30F:<20	4MGS	<0.8 m/s
Havva (10)	2020	Turkey	COPD	219	66.9 ± 10.1	-	EWGSOP2010	14.6%	BIA	M:<10.37F:<9.84	HGS	M:<28.75F:<14.25	6MWD	<0.8 m/s
Mingming Deng (34)	2022	China	COPD	235	64.4 ± 10.7	-	AWGS2019	35.3%	BIA	M:<7.0F: <5.7	HGS	M: <28F: <18	6MWD	
Qi Jiang (35)	2024	China	COPD	1,429	-	543 (35.69)	EWGSOP2	17.2%	DXA (ALM/ BMI)	M:<0.789F: <0.512	-	-	400 m walk test	
Sarah E Jones (36)	2025	UK	COPD	622	-	-	EWGSOP2010	14.5%	BIA	M:≤8.50F:≤5.75	HGS	M:<30F:<20	4MGS	<0.8 m/s
Dong-Won Lee (37)	2016	Korea	COPD	858	-	226 (79.0)	AWGS2014	33.3%	DXA	M:<7.0F: <5.4	-	-	-	-
Dong-Won Lee (38)	2022	Korea	COPD	704	-	-	-	13.9%	DXA	M/F: 0.774	-	-	-	-
Panita Limpawattana (22)	2017	Thailand	COPD	121	70 ± 9.0	112 (92.6)	AWGS2014	24.0%	DXA	M:<7.0F: <5.4	HGS	M:<26F:<18	-	-
Baiyang Lin (39)	2021	China	COPD	73	73.2 ± 9.5	59(80.8)	AWGS2014	38.4%	DXA	M:<7.0F: <5.4	HGS	M:<26F:<18	4MGS	<0.8 m/s
Nathalie (40)	2022	Mexico	COPD	185	72.2 ± 8.4	102(55.1)	EWGSOP2	42.2%	DXA	M:<7F: <6	HGS	M:<27F:<16	6MWD	-
Vitalii Poberezhets (41)	2021	Ukraine	COPD	190	66.1 ± 10.5	46(95.8)	EWGSOP2	25.3%	DXA	M:<7.0F: <5.5	HGS	M:<27F:<16	4MGS	≤0.8 m/s
Yogesh M (42)	2023	India	COPD	111	-	-	AWGS2019	52.3%	BIA	M:<7.0F: <5.5	HGS	M:<27F:<20	-	-
Yogesh M (43)	2024	India	COPD	160	48.0 ± 5.0	-	EWGSOP2	61.9%	BIA	M:<7.0F: <5.5	HGS	M:<27F:<20	-	-
Maria Tsekoura (44)	2020	Greece	COPD	69	-	-	EWGSOP2	24.6%	BIA	M:≤7F: ≤5.5	HGS	M:<27F:<16	4MGS	<0.8 m/s
Kohei Fujita, MD (45)	2022	Japan	IPF	56	73.1 ± 7.7	49(87.5)	AWGS 2019	39.3%	BIA	M:<7.0F: <5.7	HGS	M:<28F:<18	10-m corridor	<1.0 m/s
Kohei Fujita, MD (46)	2022	Japan	IPF	49	73.0 ± 7.7	44(89.8)	AWGS 2019	36.7%	BIA	M:<7.0F: <5.7	HGS	M:<28F:<18	10-m corridor	<1.0 m/s
Masatoshi Hanada (48)	2022	Japan	ILD	78	71(67–77)	-	AWGS 2019	32.1%	BIA	M:<7.0F: <5.7	HGS	M:<28F:<18	6MWD	<1.0 m/s
Jeeshitha M (49)	2024	India	ILD	32	48.0 ± 14.8	8(25)	-	21.9%	BIA	M:<7.0F: <5.7	-	-	4MGS	<0.8 m/s
Hirotsugu Ohkubo (47)	2022	Japan	IPF	54	73.6 ± 7.9	-	-	38.9%	BIA	M:<7.0F: <5.7	HGS	M:<28F:<18	10-m corridor	<1.0 m/s

### 3.3 Assessment methods for sarcopenia in end-stage lung disease patients

The included studies were evaluated based on sarcopenia-specific diagnostic criteria (Table 1). The diagnoses were established through the measurement of low lean muscle mass (LMM) and low muscle strength (LMS). Among these studies, three used only LMM as the diagnostic criterion, while 21 studies combined it with low physical performance (LPP) and/or LMS. LMM, LMS, and LPP were assessed using various methods and cutoff values across these studies. Muscle mass was quantified using dual-energy X-ray absorptiometry (DXA) and bioelectrical impedance analysis (BIA). Muscle strength was evaluated through handgrip strength (HGS) measurement. Physical performance was assessed using either the 6-min walk test (6MWT) or the 4-meter gait speed test (4MGS). The studies primarily relied on the thresholds for muscle mass, strength, and physical performance recommended by the Asian Working Group for Sarcopenia (AWGS) (17, 50) and the European Working Group on Sarcopenia in Older People (EWGSOP) (18, 51).

### 3.4 Prevalence of sarcopenia in end-stage lung disease patients

**Figure 2:**
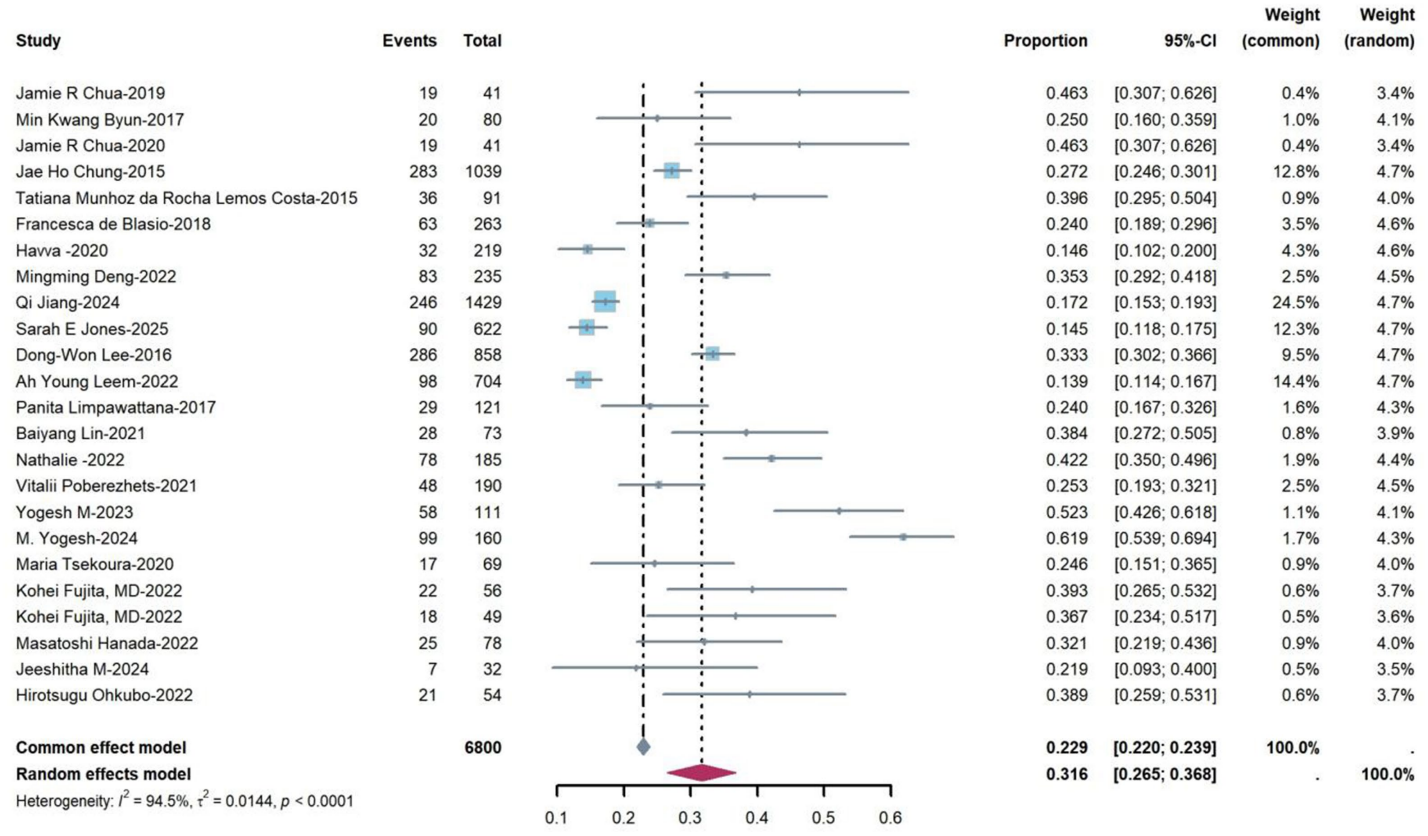
Prevalence of sarcopenia in end-stage pulmonary disease patients based on a random efforts model.

Twenty-four studies reported the prevalence of sarcopenia among patients with end-stage lung disease (range from 13.9 to 61.9%). Heterogeneity testing revealed an I^2^ statistic of 94.5% (*p* < 0.001), prompting the use of a random-effects model for the meta-analysis. The forest plot in Figure 2 illustrates the prevalence rates and their corresponding confidence intervals. The meta-analysis yielded a pooled prevalence of 31.6% (95% CI: 26.5–36.8%).

#### 3.4.1 Subgroup analysis

This systematic review conducted subgroup analyses stratified by age, sex, diagnostic criteria, measurement tools, geographic region, and disease type. The results demonstrated a higher prevalence in individuals aged ≥70 years (36%) compared to those < 70 (33%). Male patients have a higher incidence rate (33%) than female patients (11%). When assessed by diagnostic criteria, the AWGS2019 criteria yielded a higher prevalence rate (34%) than the EWGSOP2 (31%), AWGS2014 (31%), and EWGSOP2010 (22%) criteria. The use of bioelectrical impedance analysis (BIA) as a measurement tool was associated with a higher prevalence (34%) compared to dual-energy X-ray absorptiometry (DXA; 28%). Geographically, North America exhibited the highest prevalence (42%), followed by Asia (34%) and Europe (23%). Among disease subtypes, idiopathic pulmonary fibrosis (IPF) showed a greater prevalence (38%) than chronic obstructive pulmonary disease (COPD; 31%) and interstitial lung disease (ILD; 28%). All the aforementioned differences were statistically significant (*p* < 0.01; see Table 2).

**Table 2:** Meta-analysis of the prevalence in different subgroups.

Subgroup	Numbers of studies	Heterogeneity		Model	Rate (95% CI)	Test for subgroup difference
		I^2^ (%)	*p*			
Age						
<70	11	92.6	< 0.001	Random	0.33 (0.25, 0.40)	χ^2^ = 4.559, df = 1 (*p*<0.05)
≥70	7	55.4	0.036	Random	0.36 (0.30, 0.41)	
Sex						
Male	13	97.3	< 0.001	Random	0.33 (0.17, 0.49)	χ^2^ = 619, df = 1 (*p*<0.01)
Female	13	61.5	0.002	Random	0.11 (0.06, 0.17)	
Criteria						
EWGSOP2010	5	85.8	< 0.001	Random	0.22 (0.15, 0.29)	χ^2^ = 63.96, df = 3 (*p*<0.01)
EWGSOP2	5	97.6	< 0.001	Random	0.34 (0.18, 0.50)	
AWGS2014	3	67.2	0.047	Random	0.31 (0.24, 0.38)	
AWGS2019	5	63.9	0.026	Random	0.39 (0.32, 0.47)	
Assessment method						
BIA	13	92.6	< 0.001	Random	0.34 (0.25, 0.43)	χ^2^ = 36.09, df = 1 (*p*<0.01)
DXA	11	95.3	< 0.001	Random	0.28 (0.22, 0.33)	
Region						
Asia	17	95.2	< 0.001	Random	0.34 (0.28, 0.40)	χ^2^ = 27.75, df = 2 (*p*<0.01)
Europe	6	86.8	< 0.001	Random	0.23 (0.17, 0.29)	
North America	1	NA^a^	NA^a^	Random	0.42 (0.35, 0.49)	
Disease						
COPD	19	95.5	< 0.001	Random	0.31 (0.26, 0.36)	χ^2^ = 43.16, df = 2 (*p*<0.01)
IPF	3	0.0	0.958	Random	0.38 (0.31, 0.46)	
ILD	2	21.8	0.258	Random	0.28 (0.19, 0.38)	

I^2^, I-square heterogeneity statistic; P, *p* value; χ^2^, χ^2^ statistic; CI, confidence interval; NA, not applicable; a, Due to the extreme values [Rate (95% CI) <0], we use the metaprop method for analysis. Heterogeneity test results in this method were not shown when the number of subgroup studies is 3 or less.

#### 3.4.2 Sensitivity analysis

**Figure 3:**
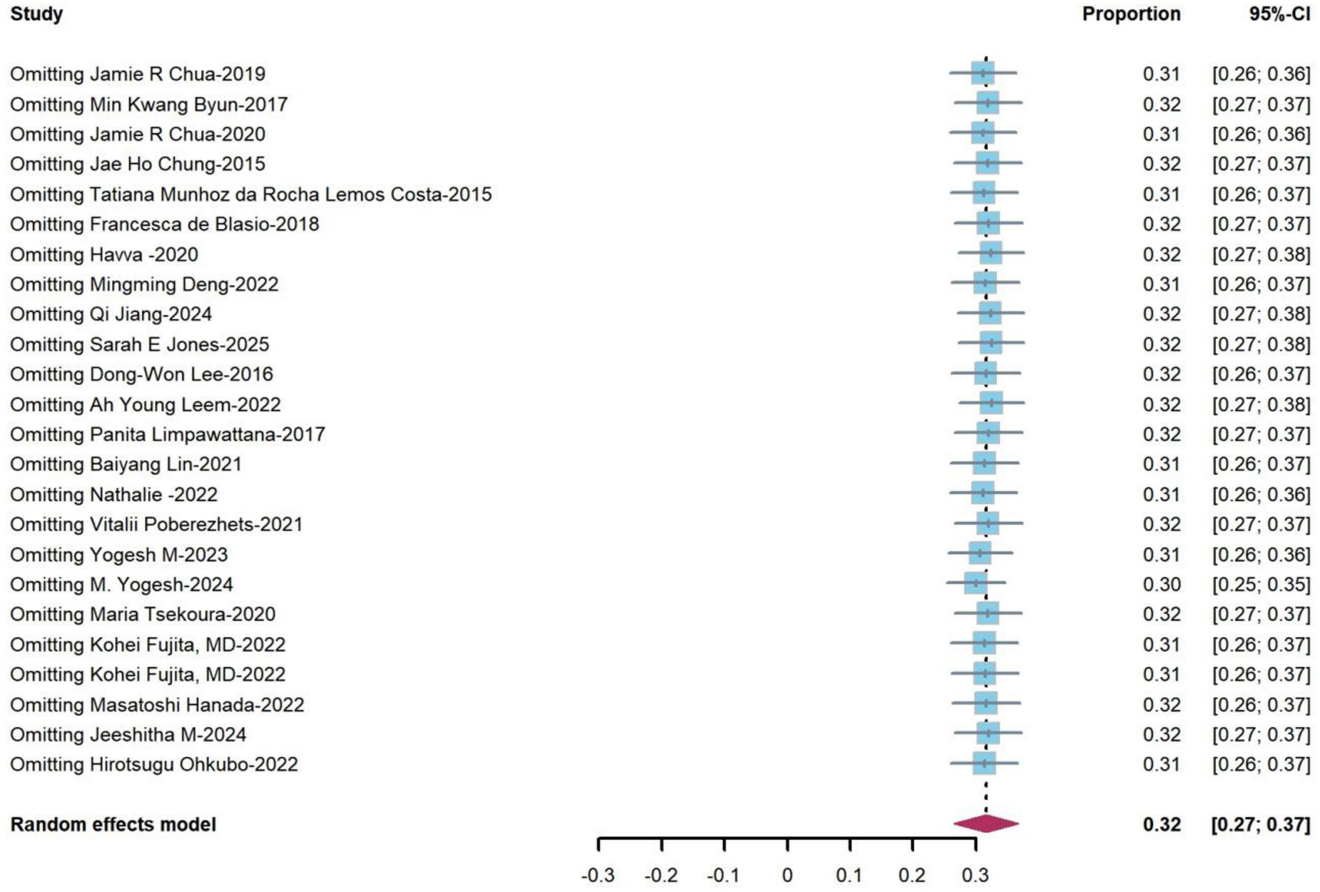
Sensitivity analysis of the studies reporting the prevalence. CI, confidence interval.

Sensitivity analysis was conducted using the leave-one-out method, and the results demonstrated robust data stability. As illustrated in Figure 3, the pooled effect size exhibited only minimal variations upon the exclusion of any individual dataset.

### 3.5 Factors correlated with sarcopenia

The main factors associated with sarcopenia in end-stage lung disease patients include age (22, 29, 34, 39, 41), particularly in the elderly population over 75 years old (22), low BMI (22, 29, 32, 34, 39, 44), and elevated systemic inflammatory markers such as hsTNF-*α* and IL-6 (39). De Blasio et al. (33) reported that 58.7% of COPD patients with sarcopenia also suffered from malnutrition (33). Qi et al. (35) found that the Dietary Inflammatory Index (DII) was associated with sarcopenia (OR: 2.37 [1.26, 4.48]). In the study by Jones et al. (36), ELD patients with sarcopenia exhibited more significant airflow limitation (manifested as reduced forced expiratory volume in 1 s[FEV1]), along with varying degrees of decline in physical activity levels, bodily function, and exercise endurance. Hirotsugu et al. (47) also observed poorer physical activity levels (*r* = −0.62) and fewer daily steps (*r* = −0.37) in patients with sarcopenia. A study (47) indicated that sarcopenia is negatively correlated with forced vital capacity (FVC; *r* = −0.51).

Whether considered independently [including both high BMI (22) and low BMI (29)] or as part of the BODE index (BMI, airflow obstruction, dyspnea, and exercise capacity) (10, 32), it is associated with sarcopenia. When analyzing psychological factors, Hirotsugu et al. (47) found that sarcopenia was positively correlated with anxiety and depression (*r* = 0.31 and *r* = 0.28, respectively). Additionally, in terms of muscle-related factors, sarcopenia in end-stage lung disease patients is also linked to the rectus femoris cross-sectional area (RFCSA), rectus femoris thickness (RFthick) (34), and skeletal muscle mass index (SMMI) (44). Relevant information can be found in the Supplementary Table 2.

### 3.6 Sarcopenia’s impact on clinical outcomes

Sarcopenia is associated with adverse clinical outcomes in patients with end-stage lung disease. Studies by Tatiana et al. (32), Havva (10), and Qi Jiang (35) demonstrated that patients with sarcopenia had poorer prognoses and higher all-cause mortality. Four studies (22, 34, 45, 47) found that sarcopenia exacerbated dyspnea severity and disease progression (22). Two studies (38, 42) indicated that sarcopenia increased the risk of cardiovascular and renal comorbidities. Additionally, one study (43) reported that sarcopenia was linked to prolonged hospitalization (>10 days) and higher 6-month readmission rates. Furthermore, two studies (10, 34) revealed that sarcopenia significantly impaired patients’ quality of life. Relevant information can be found in the Supplementary Table 2.

## 4 Discussion

ELD encompasses a range of critically debilitating conditions marked by severely impaired pulmonary function, diminished quality of life, and a poor prognosis. The presence of sarcopenia in these patients can further exacerbate their health status. However, owing to heterogeneity in study designs and patient populations, the reported prevalence of sarcopenia in patients with end-stage lung disease varied across studies included in this systematic review. To provide clinicians with robust evidence, we performed a meta-analysis of 24 studies reporting sarcopenia prevalence in this population. The pooled estimate revealed that 31.6% (95% CI: 26.5–36.8%) of end-stage lung disease patients were affected by sarcopenia, a finding that remained robust in sensitivity analyses. The pooled analysis showed a sarcopenia prevalence of 31.6% (95% CI: 26.5–36.8%) among ELD patients, with sensitivity analysis confirming the robustness of these findings. Numerous studies have reported a notably higher prevalence of sarcopenia (ranging from 10 to 39%) in patients with end-stage lung diseases, such as COPD, ILD and IPF (19–21, 45, 52). This evidence underscores the critical need to recognize and manage sarcopenia as a core comorbidity in this vulnerable population.

Subgroup analyses have revealed significant variability in the prevalence of sarcopenia across various age groups, genders, diagnostic criteria, measurement tools, geographic regions, and disease categories. Consistent with previous findings (53), the incidence of sarcopenia increases with age, reflecting the interplay of multiple physiological and disease-specific mechanisms. Chronic hypoxia and systemic inflammation in ELD patients activate the ubiquitin-proteasome system (UPS), accelerating muscle protein degradation and inducing an “accelerated aging” phenotype (54, 55). Studies indicate (19) that ELD patients, especially those with COPD, experience a shift in muscle fiber composition from type I to type II fibers (56), which likely contributes to dyspnea symptoms and impaired exercise performance (e.g., reduced endurance and exercise capacity) (57). Further clarification of the molecular and cellular mechanisms underlying sarcopenia pathogenesis in ELD populations will aid in the development of optimized assessment tools, allowing for more precise diagnosis, severity stratification, and personalized intervention strategies for this condition.

A significant sex difference in the prevalence of sarcopenia among ELD patients was observed in the overall population, with males exhibiting a higher rate than females (33% vs. 11%, *p* < 0.05). Research indicates (58) that ELD predominantly affects males due to factors such as smoking and occupational/environmental exposures. Previous studies (59) similarly report notable heterogeneity in sarcopenia incidence between genders across ELD populations. However, investigations by Awano (60) and Li (21) demonstrate that while male patients constitute the majority of ELD cohorts, no statistically significant difference in sarcopenia prevalence exists between male and female ELD patients. These findings underscore the need for further research to comprehensively characterize gender-specific epidemiological patterns and elucidate potential mechanistic pathways underlying observed sex-related disparities.

Assessment of sarcopenia prevalence in ELD populations is significantly influenced by variations in diagnostic criteria and musculoskeletal measurement tools. All included studies in this review adhered to existing sarcopenia consensus definitions, including EWGSOP 2010, EWGSOP2, AWGS 2014, and AWGS 2019. Firstly, discrepancies exist in the selection of definitions and core indicators, particularly regarding muscle mass assessment, where various criteria employ different measurement methodologies and thresholds. Inconsistencies may arise between measurements obtained via DXA and BIA, as BIA results are susceptible to fluctuations in hydration status, whereas DXA offers greater precision albeit at a higher cost. Regarding threshold settings: The Asian criteria (AWGS) (17) may establish stricter muscle mass thresholds (e.g., <7.0 kg/m^2^ for males, <5.7 kg/m^2^ for females), whereas European criteria (18) (EWGSOP) adopt slightly more lenient thresholds. Regarding muscle strength: Handgrip strength thresholds vary across different criteria. For example, AWGS defines abnormal handgrip strength as <28 kg for males and <18 kg for females, while EWGSOP may employ sex-stratified thresholds. Regarding physical performance: The inclusion or exclusion of gait speed tests (e.g., 6-meter walk) or balance assessments directly impacts diagnostic outcomes. Secondly, diagnostic algorithm complexity varies: EWGSOP requires concurrent abnormalities in muscle mass reduction plus either strength or functional decline for confirmation, whereas AWGS categorizes sarcopenia into “probable,” “confirmed,” and “severe” stages. Finally, heterogeneity exists in measurement tools and methodologies. While DXA measures total-body muscle mass, it cannot differentiate intramuscular fat infiltration. Variations in dynamometer models and testing protocols (e.g., seated vs. standing posture) may introduce measurement biases. For instance, Yogesh et al.’s study (42) (AWGS 2019) reported a 52.3% sarcopenia prevalence in ELD patients using BIA, whereas Jiang et al.’s research (35) (EWGSOP2) found a 17.2% prevalence using DXA. The lack of uniformity in diagnostic criteria and musculoskeletal assessment tools contributes to significant variability in sarcopenia prevalence estimates. Future efforts should prioritize the development of standardized definitions (e.g., disease-specific consensus guidelines) and more precise evaluation tools (e.g., AI-assisted muscle imaging analytics) to enhance interstudy comparability and diagnostic accuracy.

Our analysis revealed regional disparities in the prevalence of eldery sarcopenia, with the highest rate reported in North America (42%), followed by Asia (34%) and Europe (23%). These estimates contrast with those from two prior studies conducted in North America (61) and Europe (62), which reported rates of 7 and 48%, respectively. The heterogeneity in prevalence may be attributed to several factors. In North America, the high prevalence might be linked to the high burden of metabolic disorders—such as obesity, diabetes, and cardiovascular disease—where the co-occurrence of low physical activity and obesity (sarcopenic obesity) may exacerbate muscle loss and metabolic dysfunction (63). Conversely, several European countries (e.g., the Netherlands, Sweden, Belgium) have implemented early screening and intervention systems targeting geriatric health, including community-based programs combining nutrition and exercise, which may help mitigate sarcopenia progression (64). Although Asian countries (e.g., China, Japan) are experiencing the most rapid population aging globally, systematic screening and prevention strategies for sarcopenia remain underdeveloped in the region (65). Notably, the geographic distribution of included studies was highly uneven, with most evidence originating from Asian and European populations. The body of evidence for North America derives solely from a single study conducted in Mexico (40), constituting a significant gap in geographic representation. Given the considerable heterogeneity in ethnic background, lifestyle, and dietary habits among North American countries (e.g., Mexico, the United States, and Canada), findings from one study cannot be generalized to the entire region. This imbalance may introduce selection bias and limits the external validity of our geographic comparisons. Therefore, further high-quality studies from diverse geographic settings are needed to validate the observed regional differences in sarcopenia prevalence and clarify their underlying causes. Such efforts will be crucial for developing targeted prevention and management strategies globally.

This study also demonstrated significant differences in the prevalence of sarcopenia across various types of ELD. IPF had the highest prevalence rate at 38%, followed by COPD at 31%, both figures exceeding previously reported rates of 26 and 27%, respectively (21, 53). ILD exhibited the lowest prevalence at 28%. Potential reasons for these disparities include the following: IPF is characterized by the persistent overexpression of pro-inflammatory cytokines, such as IL-6, TNF-*α*, and TGF-*β*. These not only promote pulmonary fibrosis but also accelerate muscle protein degradation by activating the ubiquitin-proteasome system (UPS) and inhibiting the mTOR signaling pathway, thereby suppressing protein synthesis (66). On the other hand, inflammation associated with COPD predominantly involves neutrophilic infiltration (67), whereas ILD presents with heterogeneous inflammatory profiles depending on the subtype (e.g., sarcoidosis, hypersensitivity pneumonitis) (68), generally with lower systemic inflammatory intensity compared to IPF. Patients with IPF experience earlier and more prolonged hypoxemia, especially during exertion (69), whereas COPD patients typically show intermittent hypoxia (4). Chronic hypoxia suppresses mitochondrial oxidative phosphorylation, leading to muscle lactate accumulation, insufficient ATP synthesis, and accelerated muscle fiber atrophy (70).

Beyond the factors analyzed in subgroup analyses, this systematic review revealed that body mass index (BMI), systemic inflammatory markers, the dietary inflammatory index (DII), physical activity levels, and related psychological factors are all associated with sarcopenia in patients with ELD. The relationship between BMI and the prevalence of sarcopenia presents conflicting research findings, indicating a “dual effect” of BMI. A low BMI, associated with malnutrition, is directly linked to sarcopenia (29), reflecting muscle wasting due to insufficient energy-protein intake. On the other hand, a high BMI, related to obesity, also elevates the risk (22), possibly due to pro-inflammatory cytokines derived from adipocytes (e.g., IL-6) that induce muscle insulin resistance and contribute to a “sarcopenic obesity” phenotype. Elevated DII scores correlate with increased levels of pro-inflammatory cytokines (e.g., IL-6, TNF-*α*, CRP) and elevated hsTNF-α (29, 35), suggesting that chronic inflammation may accelerate muscle protein degradation through the activation of the (Ubiquitin-Proteasome System, UPS) and inhibit muscle stem cell proliferation.

A study (36) included in this analysis revealed that patients with sarcopenia exhibited significantly lower levels of physical activity compared to those without sarcopenia (*p* < 0.01). One potential mechanism underlying this association may be that physical activity reduces levels of pro-inflammatory cytokines such as IL-6 and TNF-α, while elevating anti-inflammatory factors like IL-10, thereby attenuating inflammation-mediated muscle proteolysis (4, 71). Consequently, increasing physical activity is essential for ameliorating sarcopenia. In support of this, Constantin et al. (72) demonstrated that an 8-week resistance training program led to improvements in both muscle mass and strength. Additionally, patients with anxiety and depression often exhibit hyperactivity of the hypothalamic–pituitary–adrenal (HPA) axis, resulting in chronically elevated cortisol levels (73). Elevated cortisol may contribute directly to muscle loss by suppressing insulin-like growth factor-1 (IGF-1) signaling and activating proteolytic pathways in skeletal muscle (74).

It must be acknowledged that this study did not include smoking—a critical exposure factor for ELD. Despite the fact that smoking is a major risk factor for ELD, research findings regarding its association with sarcopenia are inconsistent. One study confirmed a significant correlation between smoking and sarcopenia (32), whereas two other studies found no statistically significant association (22, 75). Jone et al. (36) further reported that there were no significant differences in smoking status (current, former, or never smokers) between COPD patients with and without sarcopenia, suggesting that smoking may indirectly affect muscle function through alternative mechanisms, such as exacerbating pulmonary dysfunction (19).

Sarcopenia significantly contributes to adverse clinical outcomes in patients with end-stage lung disease, increasing mortality, reducing quality of life, and accelerating respiratory functional decline. Corticosteroids, frequently prescribed during acute exacerbations of COPD, ILD, and IPF, can aggravate muscle atrophy (62, 76–78). Studies demonstrate that even brief glucocorticoid exposure enhances proteolytic pathways via activation of the UPS and suppression of the mTOR signaling axis, while concurrently impairing protein synthesis—collectively hastening the progression of sarcopenia (54, 79). This effect is particularly detrimental in patients with pre-existing malnutrition, systemic inflammation, and physical inactivity, further compromising muscle function and long-term prognosis (6). The pathophysiological interplay between sarcopenia and ELD is characterized by a self-perpetuating systemic-organ vicious cycle. Consequently, early screening and multimodal intervention for sarcopenia represent a pivotal therapeutic target to attenuate disease progression in ELD.

This review has several potential limitations. Firstly, all the included studies were cross-sectional in design, which precludes causal inferences and results in less rigorous exploration of influencing factors. Secondly, due to variations in measurement tools, diagnostic criteria, sampling methods, and sample sources across studies, significant heterogeneity persisted despite subgroup analyses. This may compromise the accuracy of the findings and warrants further investigation. Additionally, in this review, the diagnostic criteria adopted by each included study were independently defined by the respective authors, potentially referencing different guidelines without considering cultural and population-specific adaptations, further contributing to heterogeneity. Finally, due to resource constraints, we were unable to include unpublished literature or data. Therefore, more in-depth research is needed to investigate sarcopenia in patients with ELD.

## 5 Conclusion

Sarcopenia is highly prevalent in patients with end-stage lung disease (ELD), with a pooled prevalence of 31.6% (95% CI: 26.5–36.8), underscoring its considerable burden in this population and calling for heightened clinical attention. Our analysis reveals marked heterogeneity in sarcopenia manifestations among ELD patients, influenced by multiple factors including divergent assessment methods, lack of diagnostic standardization, and age-related physiological decline.

This systematic review synthesizes current evidence to inform screening and management strategies for sarcopenia in ELD, while highlighting the critical challenge posed by inconsistent diagnostic criteria. To address this issue, we propose the development of structured and standardized disease-specific diagnostic algorithms that integrate functional and morphological measures to improve accuracy in identification and targeting of interventions. Furthermore, we emphasize the urgent need for harmonized definitions and evaluation frameworks to enhance comparability across studies. Moving forward, priority should be given to prospective clinical trials designed to evaluate integrated care pathways that concurrently target sarcopenia and respiratory function. Such studies will be essential for generating robust evidence to guide future clinical practice recommendations.

## Data Availability

The original contributions presented in the study are included in the article/Supplementary material, further inquiries can be directed to the corresponding author.
