# Supplementary material for: Sarcopenia in end-stage lung disease patients: a systematic review and meta-analysis
Source: Front Med (Lausanne). 2025 Oct 3;12:1640027. doi: 10.3389/fmed.2025.1640027 (PMC12531037; doi:10.3389/fmed.2025.1640027)
Supplement: Supplementary file 1 [file Data_Sheet_1.docx]

JBI Critical Appraisal Checklist for Analytical Cross Sectional Studies

Supplementary Table 1 Quality assessment of the included studies

| First author | Year | Country | Disease  type | ① | ② | ③ | ④ | ⑤ | ⑥ | ⑦ | ⑧ |
| --- | --- | --- | --- | --- | --- | --- | --- | --- | --- | --- | --- |
| Jamie R Chua | 2019 | Philippines | COPD | Yes | Yes | Yes | Yes | No | No | Yes | Yes |
| Min Kwang Byun | 2017 | South Korea | COPD | Yes | Yes | Yes | Yes | No | No | Yes | Yes |
| Jamie R Chua | 2020 | Philippines | COPD | Yes | Yes | Yes | Yes | Yes | Yes | Yes | Yes |
| Jae Ho Chung | 2015 | South Korea | COPD | Yes | Yes | Yes | Yes | Unclear | Unclear | Yes | Yes |
| Tatiana Munhoz | 2015 | Brasil | COPD | Yes | Yes | Yes | Yes | Yes | Unclear | Yes | Yes |
| Francesca de Blasio | 2018 | Italy | COPD | Yes | Yes | Yes | Yes | Unclear | Unclear | Yes | Yes |
| Havva | 2020 | Turkey | COPD | Yes | Yes | Yes | Yes | Yes | Unclear | Yes | Yes |
| Mingming Deng | 2022 | China | COPD | Yes | Yes | Yes | Yes | Yes | Unclear | Yes | Yes |
| Qi Jiang | 2024 | China | COPD | Yes | Yes | Yes | Yes | Yes | Yes | Yes | Yes |
| Sarah E Jones | 2025 | UK | COPD | Yes | Yes | Yes | Yes | Unclear | Unclear | Yes | Yes |
| Dong-Won Lee | 2016 | Korea | COPD | Yes | Yes | Yes | Yes | Yes | Yes | Yes | Yes |
| Ah Young Leem | 2022 | Korea | COPD | Yes | Yes | Yes | Yes | No | No | Yes | Yes |
| Panita Limpawattana | 2017 | Thailand | COPD | Yes | Yes | Yes | Yes | No | No | Yes | Yes |
| Baiyang Lin | 2021 | China | COPD | Yes | Yes | Yes | Yes | Yes | No | Yes | Yes |
| Nathalie | 2022 | Mexico | COPD | Yes | Yes | Yes | Yes | No | No | Yes | Yes |
| Vitalii Poberezhets | 2021 | Ukraine | COPD | Yes | Yes | Yes | Yes | Unclear | Unclear | Yes | Yes |
| Yogesh M | 2023 | India | COPD | Yes | Yes | Yes | Yes | Unclear | Unclear | Yes | Yes |
| M. Yogesh | 2024 | India | COPD | Yes | Yes | Yes | Yes | No | No | Yes | Yes |
| Maria Tsekoura | 2020 | Greece | COPD | Yes | Yes | Yes | Yes | Unclear | Unclear | Yes | Yes |
| Kohei Fujita, MD | 2022 | Japan | IPF | Yes | Yes | Yes | Yes | No | No | Yes | Yes |
| Kohei Fujita, MD | 2022 | Japan | IPF | Yes | Yes | Yes | Yes | No | No | Yes | Yes |
| Masatoshi Hanada | 2022 | Japan | IPF | Yes | Yes | Yes | Yes | No | No | Yes | Yes |
| Jeeshitha M | 2024 | India | IPF | Yes | Yes | Yes | Yes | Unclear | Unclear | Yes | Yes |
| Hirotsugu Ohkubo | 2022 | Japan | IPF | Yes | Yes | Yes | Yes | Unclear | Unclear | Yes | Yes |

Supplementary table 1 Quality assessment of the included studies

①Were the criteria for inclusion in the sample clearly defined? ②Were the study subjects and the setting described in detail?③Was the exposure measured in a valid and reliable way?.④Were objective, standard criteria used for measurement of the condition?⑤Were confounding factors identified? ⑥Were strategies to deal with confounding factors stated? ⑦Were the outcomes measured in a valid and reliable way? ⑧ Was appropriate statistical analysis used?

Supplementary Table2 Factors correlated and Clinical outcome with sarcopenia

| First author | Year | Country | Sample size | Age  (mean ± SD) | Relevant factors | | | | Clinical outcome |
| --- | --- | --- | --- | --- | --- | --- | --- | --- | --- |
|  |  |  |  |  | correlation coefficient(r) | OR(95%CI) | | |  |
| Jamie R Chua | 2019 | Philippines | 41 | 69.2±6.8 | ①peak inspiratory flow (r= -0.5791);  ②peak expiatory flow (r= -0.4475);  ③handgrip strength (r= -0.4560);  ④lower CAT score (r= -0.3422) |  | | | - |
| Min Kwang Byun | 2017 | South Korea | 80 | 68.4±8.9 |  | ①old age:1.19[1.03, 1.36];  ②low BMI:0.52[0.35, 0.78];  ③presence of previous cardiovascular disease:4.66[1.01, 18.31];  ④higher hsTNFα levels:1.99[1.04, 3.81] | | | ①severe symptoms of dyspnea  ②poorer exercise tolerance  ③ higher BODE index  ④higher hsTNFα  and IL-6 |
| Jamie R Chua | 2020 | Philippines | 41 | 69.2±6.8 | Osteoporosis(p＜0.05) | | | |  |
| Jae Ho Chung | 2015 | South Korea | 1039 | Man：64.5±9.4  Woman：64.5±10.2 |  | Man:  ①abdominal obesity: 3.66[-2.38, 5.65];  ②metabolic syndrome:1.88[1.27, 2.77];  Female:  ①abdominal obesity:5.03[1.91, 13.23] | | | - |
| Tatiana Munhoz | 2015 | Brasil | 91 | 67.4±8.7 | ①lower BMI  ②lower percentage of total body fat  ③lower total lean mass  ④the BODE quartile | | | | poor prognosis |
| Francesca de Blasio | 2018 | Italy | 263 | 68.0±9.0 | malnutrition(p＜0.05) | | | | - |
| Havva | 2020 | Turkey | 219 | 66.9±10.1 |  | | ①BMI < 30: 36.340[12.39,106.59]  ②BODE classification  BODE3-4: 5.52[1.50, 20.36]  BODE5- 6: 11.02[1.560, 77.84]  BODE7-10: 34.392[4.492, 263.308] | | ①decrease in exercise capacity  ②diminished quality of life  ④increased mortality |
| Mingming Deng | 2022 | China | 235 | 64.4±10.7 |  | | ①Age: 1.125[1.04, 1.22]  ②BMI: 0.800[0.68, 0.94]  ③RFthick: 0.216[0.146, 0.493]  ④RFcsa: 0.132[0.071, 0.198] | | ①lower quality of life  ②higher SGRQ  symptoms, activity, impact, and total scores；  ③higher BODE |
| Qi Jiang | 2024 | China | 1429 | - |  | | Dietary Inflammatory Index: 2.37[1.26, 4.48] | | higher risk of all-cause  mortality |
| Sarah E Jones | 2025 | UK | 622 | - | reduced functional performance, exercise capacity and  quality of life((p＜0.001)) | | | | - |
| Dong-Won Lee | 2016 | Korea | 858 | - |  | | | ①hincreased the risk of osteopenia: 3.23[2.13, 4.90]  ②hincreased the risk ofosteoporosis: 6.95[3.42, 14.14]  ③low BMD: 3.50[2.32, 5.28] | - |
| Ah Young Leem | 2022 | Korea | 704 | - |  | | | High ASCVD risk: 2.32[1.05, 5.15] |  |
| Panita Limpawattana | 2017 | Thailand | 121 | 70±9.0 |  | | | ①Age  ＞75 years: 5.3[1.6, 17.7]  ②Severity of COPD:  Severe: 5.7[1.3, 25.3]  ③MMRC scale: 1.9[1.3, 2.8]  ④Nonelective admission: 2.0[1.1, 3.7]  ⑤BMI (kg/m^2^)  Normal and over weight: 0.3[0.1, 0.8]  Obesity: 0.02[0.02, 0.2]  ⑥MAP(mmHg): 0.9[0.9, 0.9] |  |
| Baiyang Lin | 2021 | China | 73 | 73.2±9.5 |  | | | ①Age: 1.11[1.01, 1.23]  ②BMI(Kg/m^2^): 0.54[0.36, 0.80]  ③IL-6:1.07[1.01,1.12] |  |
| Nathalie | 2022 | Mexico | 185 | 72.2±8.4 |  | | | ①FEV1(% predicted): −6.99[−13.72, −0.26] |  |
| Vitalii Poberezhets | 2021 | Ukraine | 190 | 66.1±10.5 |  | | | ①higher level of visceral fat: 1.97[1.25, 3.10]  ②age:1.23[1.04,1.45]  ③SGRQ activity score:1.20[1.02, 1.42]  ④lower BMI: 0.28[0.13, 0.61] |  |
| Yogesh M | 2023 | India | 111 | - |  | | | ①Concealed chronic  renal failure (CRF): 4.11[1.52, 10.20]  ②Overt chronic  renal failure (CRF): 3.80[1.64, 8.61] |  |
| M. Yogesh | 2024 | India | 160 | 48.0±5.0 | - | | | - | ①Prolonged LOS (>10Days)  ②Re-Admission within 6months：  ③CAT（COPD assessment test） score≥10 |
| Maria Tsekoura | 2020 | Greece | 69 | - |  | | | ①BMI: 2.94[1.52-5.69]  ②SMMI: 28.6[32.53-251.7]  ③HGS:1.67[1.02-2.73]  ④4 m test: 0.52[0.29-0.93]  ⑤Drug: 0.66[0.46-0.94]  ⑥COPD: 0.8[0.41-0.94] |  |
| Kohei Fujita, MD | 2022 | Japan | 56 | 73.1±7.7 |  | | | 6MWD: 0.98[0.96, 0.99] | ①physical performance  ②Patient-reported outcomes(PROs): SGRQ-total score; SGRQ-activity score; SGRQ-impact score;  MRC score; HADS-depression  score |
| Kohei Fujita, MD | 2022 | Japan | 49 | 73.0±7.7 |  | | | Cr/CysC ratio: 0.85[0.73, 0.97] | ①mMRC score;  ②CAT；  ③K-BILD-breathlessness score；  ④K-BILDtotal  score |
| Masatoshi Hanada | 2022 | Japan | 78 | 71(67–77) | - | | | | - |
| Jeeshitha M | 2024 | India | 32 | 48.0±14.8 | - | | | | - |
| Hirotsugu Ohkubo | 2022 | Japan | 54 | 73.6±7.9 | ①forced vital capacity(r= −0.51)  ②CAT score(r=0.57)  ③SGRQ total score(r=0.77)  ④Hospital Anxiety and Depression Scale anxiety score(r=0.31)  ⑤Hospital Anxiety and  Depression Scale depression score(r=0.28)  ⑥6MWT(r=−0.62)  ⑦daily step count(r=−0.37) | | | ①daily step count: 0.39[−1,342, −252] | - |

sarcopenia："sarcopenias" OR "sarcopenic" OR "muscle loss"OR "muscle mass"OR "muscle waste" OR "muscle wasting" OR "skeletal muscle reduction"OR "muscle weakness" OR "muscular atrophy"

COPD：

①Pubmed N=1178

("pulmonary disease, chronic obstructive"[MeSH Terms] OR ("COPD"[Title/Abstract] OR "chronic obstructive pulmonary disease"[Title/Abstract] OR "chronic obstructive lung disease"[Title/Abstract] OR "COAD"[Title/Abstract])) AND ("Sarcopenia"[MeSH Terms] OR ("sarcopenias"[Title/Abstract] OR "sarcopenic"[Title/Abstract] OR "muscle loss"[Title/Abstract] OR "muscle mass"[Title/Abstract] OR "muscle waste"[Title/Abstract] OR "muscle wasting"[Title/Abstract] OR "skeletal muscle reduction"[Title/Abstract] OR "muscle weakness"[Title/Abstract] OR "muscular atrophy"[Title/Abstract]))

pulmonary disease, chronic obstructive OR "COPD" OR "chronic obstructive pulmonary disease" OR "chronic obstructive lung disease" OR "COAD

COPD,” “chronic obstructive pulmonary disease,” “chronic obstructive lung disease,” “COAD,”

“chronic obstructive airway disease,”

②WOS N=1051

https://webofscience.clarivate.cn/wos/alldb/summary/b2dc2496-61b5-42a8-a63e-d5089dbc2bb5-014d8987a6/relevance/1

③Embase N=2672

#1 'chronic obstructive lung disease'/exp OR 'chronic obstructive lung disease' OR copd:ab,ti OR 'chronic obstructive lung disease':ab,ti OR coad:ab,ti OR 'chronic obstructive airway disease':ab,ti

#2 'sarcopenia'/exp OR 'sarcopenia' OR sarcopenias:ab,ti OR sarcopenic:ab,ti OR 'muscle loss':ab,ti OR 'muscle mass':ab,ti OR 'muscle waste':ab,ti OR 'muscle wasting':ab,ti OR 'skeletal muscle reduction':ab,ti OR 'muscle weakness':ab,ti OR 'muscular atrophy':ab,ti

#1 AND #2

④Cochrane library N=395

⑤CINAHL N=348

⑥Scopus N=213

TITLE-ABS-KEY(sarcopeni* OR muscle loss OR muscle mass OR muscle waste OR muscle wasting OR skeletal muscle reduction OR muscle weakness OR muscular atrophy ) AND TITLE-ABS-KEY(pulmonary disease, chronic obstructive OR COPD OR chronic obstructive pulmonary disease OR chronic obstructive lung disease OR COAD)

Idiopathic Pulmonary Fibrosis or interstitial lung disease or idiopathic lung disease

①Pubmed N=296

**(("Idiopathic Pulmonary Fibrosis"[Mesh]) OR ((interstitial lung disease[Title/Abstract]) OR (idiopathic lung disease[Title/Abstract]))) AND (("Sarcopenia"[Mesh]) OR (((((((((sarcopenias[Title/Abstract]) OR (sarcopenic[Title/Abstract])) OR (muscle loss[Title/Abstract])) OR (muscle mass[Title/Abstract])) OR (muscle waste[Title/Abstract])) OR (muscle wasting[Title/Abstract])) OR (skeletal muscle reduction[Title/Abstract])) OR (muscle weakness[Title/Abstract])) OR (muscular atrophy[Title/Abstract])))**

②WOS N=125

**https://webofscience.clarivate.cn/wos/alldb/summary/cfe4569e-1bee-4795-a09d-a1c3655251fd-014d89ab7a/relevance/1**

③Embase N=920

#1 'idiopathic pulmonary fibrosis'/exp OR 'idiopathic pulmonary fibrosis' OR (idiopathic AND pulmonary AND ('fibrosis'/exp OR fibrosis)) OR 'interstitial lung disease':ab,ti OR 'idiopathic lung disease':ab,ti

#2 'sarcopenia'/exp OR 'sarcopenia' OR sarcopenias:ab,ti OR sarcopenic:ab,ti OR 'muscle loss':ab,ti OR 'muscle mass':ab,ti OR 'muscle waste':ab,ti OR 'muscle wasting':ab,ti OR 'skeletal muscle reduction':ab,ti OR 'muscle weakness':ab,ti OR 'muscular atrophy':ab,ti

#1 AND #2

④Cochrane library N=29

⑤CINAHL N=106

⑥Scopus 0篇

Pulmonary Hypertension; lung arterial hypertension

①Pubmed N=121

(("Hypertension, Pulmonary"[Mesh]) OR ((Pulmonary Hypertension[Title/Abstract]) OR (lung arterial hypertension[Title/Abstract]))) AND (("Sarcopenia"[Mesh]) OR (((((((((sarcopenias[Title/Abstract]) OR (sarcopenic[Title/Abstract])) OR (muscle loss[Title/Abstract])) OR (muscle mass[Title/Abstract])) OR (muscle waste[Title/Abstract])) OR (muscle wasting[Title/Abstract])) OR (skeletal muscle reduction[Title/Abstract])) OR (muscle weakness[Title/Abstract])) OR (muscular atrophy[Title/Abstract])))

②WOS N=96

https://webofscience.clarivate.cn/wos/alldb/summary/b0eb6904-db72-4a15-94aa-c16563a6b290-014d89c85d/relevance/1

③Embase N= 464

#1'pulmonary hypertension'/exp OR 'pulmonary hypertension' OR 'hypertension, pulmonary':ab,ti OR 'lung arterial hypertension':ab,ti

#2 'sarcopenia'/exp OR 'sarcopenia' OR sarcopenias:ab,ti OR sarcopenic:ab,ti OR 'muscle loss':ab,ti OR 'muscle mass':ab,ti OR 'muscle waste':ab,ti OR 'muscle wasting':ab,ti OR 'skeletal muscle reduction':ab,ti OR 'muscle weakness':ab,ti OR 'muscular atrophy':ab,ti

#1 AND #2

④Cochrane library N=84

⑤CINAHL N=59

Hypertension, Pulmonary or Pulmonary Hypertension or lung arterial hypertension

⑥Scopus N=10

CF

(cystic fibrosis[MeSH Terms]) OR (fibrosis, cystic or mucoviscidosis or pulmonary cystic fibrosis or cystic fibrosis, pulmonary))

①Pubmed N=121

("Cystic Fibrosis"[MeSH Terms] OR ("fibrosis cystic"[Title/Abstract] OR "mucoviscidosis"[Title/Abstract] OR "pulmonary cystic fibrosis"[Title/Abstract] OR "cystic fibrosis pulmonary"[Title/Abstract])) AND ("Sarcopenia"[MeSH Terms] OR ("sarcopenias"[Title/Abstract] OR "sarcopenic"[Title/Abstract] OR "muscle loss"[Title/Abstract] OR "muscle mass"[Title/Abstract] OR "muscle waste"[Title/Abstract] OR "muscle wasting"[Title/Abstract] OR "skeletal muscle reduction"[Title/Abstract] OR "muscle weakness"[Title/Abstract] OR "muscular atrophy"[Title/Abstract]))

②WOS N=102

<https://webofscience.clarivate.cn/wos/alldb/summary/eab8a167-0df3-4ba6-b1fa-c2eda7cad19d-014d89dc91/relevance/1>

③Embase N=587

#1 'cystic fibrosis'/exp OR 'cystic fibrosis' OR 'fibrosis cystic':ab,ti OR mucoviscidosis:ab,ti OR 'pulmonary cystic fibrosis':ab,ti OR 'cystic fibrosis pulmonary':ab,ti

#2 'sarcopenia'/exp OR 'sarcopenia' OR sarcopenias:ab,ti OR sarcopenic:ab,ti OR 'muscle loss':ab,ti OR 'muscle mass':ab,ti OR 'muscle waste':ab,ti OR 'muscle wasting':ab,ti OR 'skeletal muscle reduction':ab,ti OR 'muscle weakness':ab,ti OR 'muscular atrophy':ab,ti

#1 AND #2

④Cochrane library N=65

⑤CINAHL N=79

cystic fibrosis OR fibrosis, cystic or mucoviscidosis or pulmonary cystic fibrosis or cystic fibrosis, pulmonary

⑥Scopus N=4

TITLE-ABS-KEY(sarcopeni* OR muscle loss OR muscle mass OR muscle waste OR muscle wasting OR skeletal muscle reduction OR muscle weakness OR muscular atrophy) AND TITLE-ABS-KEY(Cystic Fibrosis OR fibrosis cystic OR mucoviscidosis OR pulmonary cystic fibrosis OR cystic fibrosis pulmonary)
